# SARS-CoV-2 Proteins Induce *IFNG* in Th1 Lymphocytes Generated from CD4+ Cells from Healthy, Unexposed Polish Donors

**DOI:** 10.3390/vaccines8040673

**Published:** 2020-11-12

**Authors:** Anna Sałkowska, Iwona Karwaciak, Kaja Karaś, Jarosław Dastych, Marcin Ratajewski

**Affiliations:** 1 Laboratory of Epigenetics, Institute of Medical Biology, Polish Academy of Sciences, 93-232 Lodz, Poland; asalkowska@cbm.pan.pl (A.S.); kkaras@cbm.pan.pl (K.K.); 2 Laboratory of Transcriptional Regulation, Institute of Medical Biology, Polish Academy of Sciences, 93-232 Lodz, Poland; isachrajda@cbm.pan.pl; 3 Laboratory of Cellular Immunology, Institute of Medical Biology, Polish Academy of Sciences, 93-232 Lodz, Poland; jdastych@cbm.pan.pl

**Keywords:** SARS-CoV-2, COVID-19, Th1, Th17, IFNG, IFN-γ

## Abstract

The outbreak of the SARS-CoV-2 virus in December 2019 has caused the deaths of several hundred thousand people worldwide. Currently, the pathogenesis of COVID-19 is poorly understood. During the course of COVID-19 infection, many patients experience deterioration, which might be associated with systemic inflammation and cytokine storm syndrome; however, other patients have mild symptoms or are asymptomatic. There are some suggestions that impaired cellular immunity through a reduction in Th1 response and *IFNG* (interferon gamma) expression, as well as cross-reactivity with common cold coronaviruses, might be involved in the differential COVID-19 course. Here, we show that CD4+ cells isolated from unexposed healthy donors that were differentiated towards the Th1 lineage in the presence of SARS-CoV-2 proteins exhibited induction of *IFNG*. Interestingly, the same cells induced to differentiate towards a Th17 lineage did not exhibit changes in *IFNG* expression or Th17-related cytokines. This suggests the cellular response to SARS-CoV-2 viral proteins is primarily associated with Th1 lymphocytes and may be dependent on past infections with common cold coronaviruses or vaccinations that induce unspecific cellular responses, e.g., BCG (**B**acillus **C**almette-**G**uérin). Thus, our results might explain the high variability in the course of COVID-19 among populations of different countries.

## 1. Introduction

Severe acute respiratory syndrome coronavirus 2 (SARS-CoV-2) is an enveloped positive-strand RNA virus that has been classified within the family Coronaviridae and genus *Betacoronavirus* [1,2]. The SARS-CoV-2 virus causes COVID-19 [3] and is characterized by strong transmission ability, especially in comparison to the SARS-CoV-1 and MERS-CoV coronaviruses, which also caused global outbreaks [4,5]. The majority of patients develop mild clinical manifestations (fever, cough, shortness of breath) [6]; however, some people, such as the elderly or those with certain chronic medical conditions, are at risk for a more severe course and a higher mortality rate from COVID-19 [7]. COVID-19 is a new disease, and there is currently limited information regarding the molecular background or the factors that lead to life-threatening symptoms.

Viral infection may cause tissue damage that results from virus replication or an abnormal immunological reaction [8]. Naive CD4+ cells differentiate into different T helper (Th) subsets with distinct cytokine and transcription factor profiles and effector functions [9]. Proinflammatory Th1 and Th17 cells play a prominent role in the response to viral infections, and while Th1 cells stimulate phagocyte-dependent killing, Th17 cells may cause immunopathology through persistent immune activation [10,11,12]. T regulatory cells (Tregs) that control the inflammatory response and prevent immunopathology can also enhance viral infection upon abnormal expansion [13,14]. Therefore, complications and ultimately death that arise from SARS-CoV-2 infection might be associated with disturbances in the CD4+ T cell balance and hyperproduction of pro-inflammatory cytokines, which is known as a “cytokine storm”, leading to systemic inflammation, multiorgan failure, and death [15,16,17].

In the present study, we examined whether interactions between CD4+ cells isolated from healthy donors unexposed to SARS-CoV-2 and recombinant virus proteins in vitro result in an altered response to anti-CD3/anti-CD28-mediated polyclonal T cell activation. We found that N protein (nucleocapsid) and S protein (spike) induce *IFNG* and, to a lesser extent, *IL2* in CD4+ cells differentiated towards Th1 cells, while no effect was observed in cells differentiating into Th17 lymphocytes. This suggests the observed cellular response to SARS-CoV-2 proteins is dependent on the Th subtype. Furthermore, we observed differences in the levels of T cell response between individual donors, suggesting SARS-CoV-2 induces Th1-restricted polyclonal stimulation that might depend on the immunological status of the individual and may result from previous infections (e.g., with common cold coronaviruses) or different factors, including vaccination (e.g., BCG (**B**acillus **C**almette-**G**uérin) vaccination).

## 2. Materials and Methods

**Figure 1:**
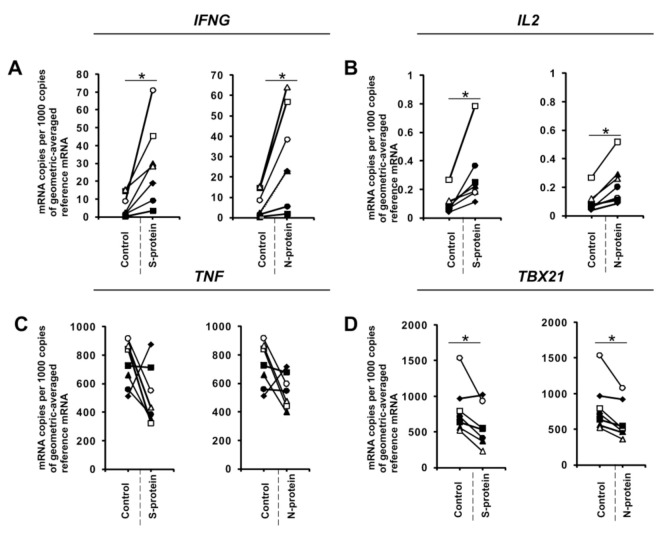
SARS-CoV-2 proteins mediate gene expression in CD4+ cells isolated from healthy, unexposed donors that were differentiated into Th1 lymphocytes. mRNA expression of *IFNG* (**A**), *IL2* (**B**), *TNF* (**C**), and *TBX21* (**D**) genes in CD4+ cells differentiated towards Th1 cells in the presence of 1 μg/mL SARS-CoV-2 proteins S (spike) and N (nucleocapsid) for 5 days determined by real-time RT-PCR and normalized to averaged reference mRNA levels of the housekeeping genes *HPRT1*, *HMBS*, and *RPL13A*. Data show individual values for seven independent donors (*n* = 7). “Control” denotes cells cultured in the absence of SARS-CoV-2 proteins for 5 days, and “S-protein” or “N-protein” denotes cells cultured in the presence of SARS-CoV-2 protein (S or N, respectively) for 5 days. Control values are the same for both conditions (S and N proteins). Straight lines connecting two points (control and given protein) identify samples from a specific donor. An asterisk indicates a statistically significant difference at *p* < 0.05. Statistical analysis was performed using Friedman repeated measures ANOVA on ranks followed by Student–Newman–Keuls post hoc test.

**Figure 2:**
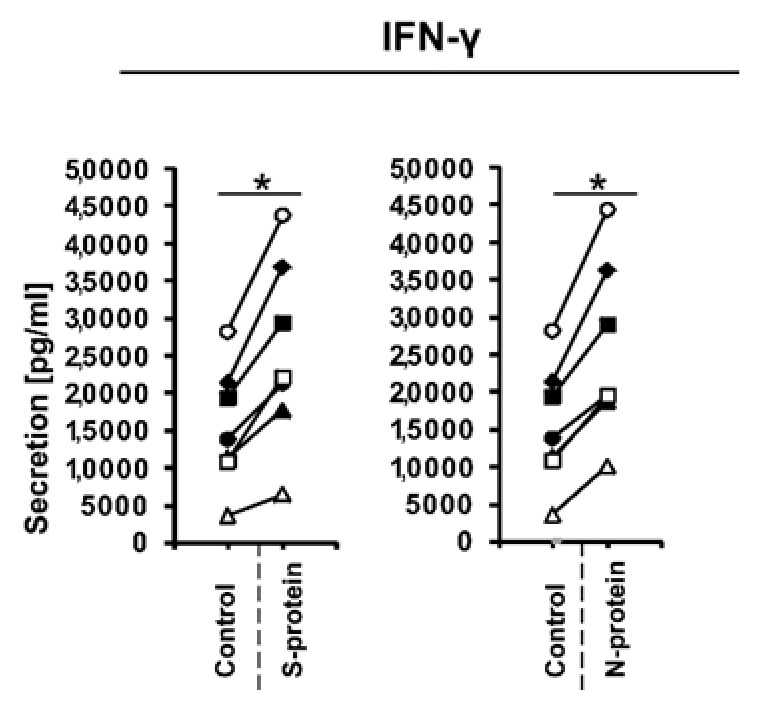
IFN-γ production in cellular supernatants of CD4+ cells isolated from healthy, unexposed donors that were differentiated into Th1 lymphocytes in the presence of 1 μg/mL SARS-CoV-2 proteins S (spike) and N (nucleocapsid) for 5 days was determined using the human IFN-gamma Quantikine ELISA Kit. Data show individual values for seven independent donors (*n* = 7). “Control” denotes cells cultured in the absence of SARS-CoV-2 proteins for 5 days, and “S-protein” or “N-protein” denotes cells cultured in the presence of SARS-CoV-2 protein (S or N, respectively) for 5 days. Control values are the same for both conditions (S and N proteins). Straight lines connecting two points (control and given protein) identify samples from a specific donor. An asterisk indicates a statistically significant difference at *p* < 0.05. Statistical analysis was performed using Friedman repeated measures ANOVA on ranks followed by Student–Newman–Keuls post hoc test.

**Figure 3:**
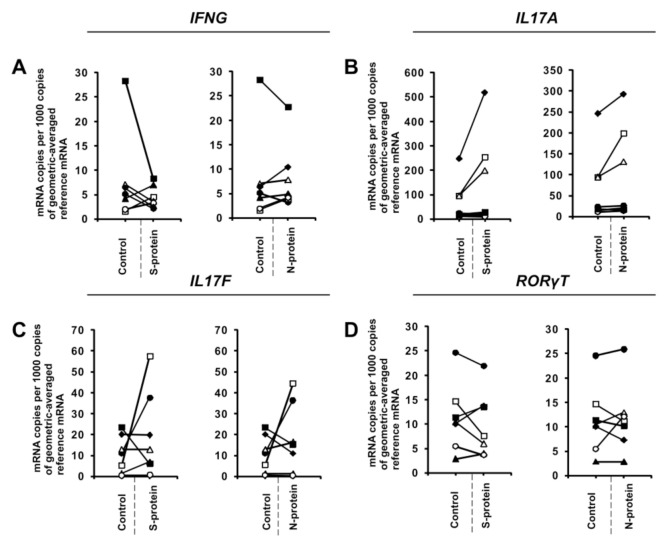
SARS-CoV-2-specific gene expression in CD4+ cells isolated from healthy, unexposed donors that were differentiated into Th17 lymphocytes. mRNA expression of *IFNG* (**A**), *IL17A* (**B**), *IL17F* (**C**), and *RORγT* (**D**) genes in CD4+ cells differentiated towards Th17 cells in the presence of 1 μg/mL SARS-CoV-2 proteins S (spike) and N (nucleocapsid) for 5 days determined by real-time RT-PCR and normalized to averaged reference mRNA levels of the housekeeping genes *HPRT1*, *HMBS*, and *RPL13A*. “Control” denotes cells cultured in the absence of SARS-CoV-2 proteins for 5 days, and “S-protein” or “N-protein” denotes cells cultured in the presence of SARS-CoV-2 protein (S or N, respectively) for 5 days. Control values are the same for both conditions (S and N proteins). Straight lines connecting two points (control and given protein) identify samples from a specific donor. Data show individual values for seven independent donors (*n* = 7).

**Figure 4:**
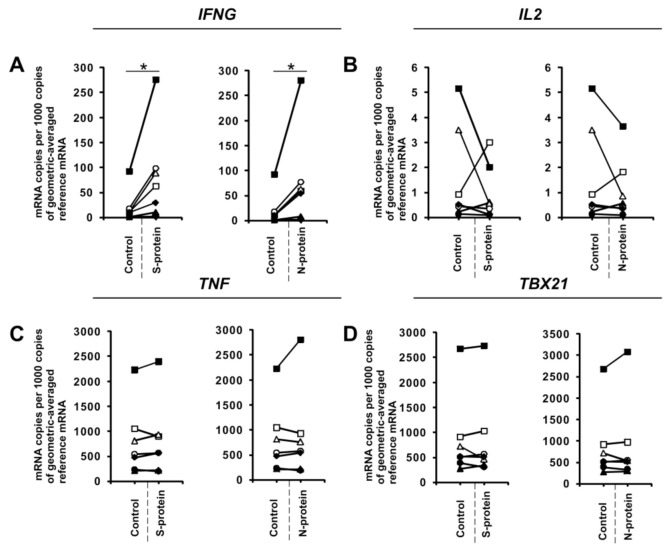
SARS-CoV-2-specific gene expression in fully differentiated Th1 cells. CD4+ cells were differentiated towards Th1 lymphocytes for 5 days, restimulated with anti-CD3 and anti-CD28 beads, and then activated with 1 μg/mL SARS-CoV-2 proteins S (spike) and N (nucleocapsid) for 24 h. mRNA expression of *IFNG* (**A**), *IL2* (**B**), *TNF* (**C**), and *TBX21* (**D**) genes in Th1 cells. Data show individual values for seven independent donors (*n* = 7). “Control” denotes cells cultured in the absence of SARS-CoV-2 proteins for 24 h, and “S-protein” or “N-protein” denotes cells cultured in the presence of SARS-CoV-2 protein (S or N, respectively) for 24 h. Control values are the same for both conditions (S and N proteins). Straight lines connecting two points (control and given protein) identify samples from a specific donor. An asterisk indicates a statistically significant difference at *p* < 0.05. Statistical analysis was performed using Friedman repeated measures ANOVA on ranks followed by Student–Newman–Keuls post hoc test.

CD4+ T cell isolation and differentiation. Peripheral blood mononuclear cells were isolated from buffy coats (purchased from the Regional Center for Blood Donation and Blood Treatment, Łódź, Poland) obtained from the blood of anonymous healthy donors from the Polish population. All donors were analyzed in the order of receipt of blood from that station from 24 June to 18 August 2020. Plasma samples were tested for the presence of anti-SARS-CoV-2 antibodies using the Cellex qSARS-CoV-2 IgG/IgM Rapid Test (Cellex Inc., Raleigh, NC, USA) and the EDI^TM^ Novel Coronavirus COVID-19 IgG ELISA Kit (Epitope Diagnostics Inc., San Diego, CA, USA). All serum samples were negative. Considering that at 93.8% specificity, 96% sensitivity, and 1% prevalence the NPV was 99.9%, all buffy coats were treated as having been obtained from unexposed individuals. The results obtained and shown in Figure 1, Figure 2 and Figure 3 are from the same 7 donors, while those shown in Figure 4 are from 7 other donors; thus, in total, cells from 14 donors were analyzed. The CD4+ fraction was isolated using CD4 M-pluriBead^®^ anti-hu (pluriSelect Life Science, Leipzig, Germany). Human Th1 cells were differentiated using the Human Th1 Cell Differentiation Kit (R and D CDK001) and were cultured in RPMI 1640 medium, supplemented with 5% FBS, 2 mM L-glutamine, 50 units/mL penicillin, 50 µg/mL streptomycin, and 50 µM 2-ME with Human Th1 Reagent 1 and Human Th1 Reagent 2 (part of the Human Th1 Cell Differentiation Kit) for 5 days. Th17 cells were generated using the protocol described by Wilson et al. [18]. CD4+ cells were cultured in Yssel’s medium containing human AB serum, anti-CD2, anti-CD3, and anti-CD28 beads (T cell activation/expansion kit from Miltenyi Biotec) as well as the following cytokines for 5 days: human IL-1β (50 ng/mL), human IL-6 (30 ng/mL), human IL-23 (10 ng/mL), and human transforming growth factor beta (TGF-β) (10 ng/mL). 

Reagents. All cytokines were purchased from PeproTech (Cranbury, NJ, USA). Recombinant 2019-nCoV S1 Protein (Active) 32-190005 and COVID-19 Nucleocapsid Protein 32-190001 were purchased from Abeomics (San Diego, CA, USA).

Activation of CD4+ cells using SARS-CoV-2 proteins. To analyze the effects of SARS-CoV-2 proteins on differentiating Th1 and Th17 cells, CD4+ cells were isolated from healthy, unexposed donors and were further differentiated into Th1 or Th17 lymphocytes for 5 days in the presence of 1 μg/mL SARS-CoV-2 proteins S (spike) and N (nucleocapsid). Subsequently, the cells and supernatants were collected for RNA isolation and ELISA assays. To analyze the effects of SARS-CoV-2 proteins on fully differentiated Th1 cells, CD4+ cells were isolated from healthy, unexposed donors and were further differentiated into Th1 cells for 5 days. After that, the cells were washed and resuspended in medium supplemented with anti-CD3 and anti-CD28 beads without cytokines, and they were treated with 1 μg/mL SARS-CoV-2 proteins S (spike) and N (nucleocapsid) for 24 h. Then the cells were collected for RNA isolation.

Real-time RT-PCR. Total RNA was extracted from cells using TRI Reagent (Molecular Research Center, Cincinnati, OH, USA) according to the manufacturer’s instructions. Next, 5 μg RNA was used to obtain cDNA using the Maxima First Strand cDNA Synthesis Kit for RT-quantitative PCR (Thermo Fisher Scientific, Waltham, MA, USA). Real-time RT-PCR was performed using SYBR Green I Master Mix on a LightCycler 480 (Roche, Basel, Switzerland) in a 384-well white plate under the following conditions: 95 °C for 5 min followed by 45 cycles of 95°C for 10 s, 60°C for 10 s, and 72°C for 20 s. Primers for *TBX21*, *RORγT*, *IL17A*, and *IL17F* were described previously [19,20,21]. Primers for *IFNG* and *TNF* were designed using Primer3 [22] and QuantPrime [23], respectively. Expression of mRNA was normalized to the geometric mean of the housekeeping genes *HPRT1*, *HMBS*, and *RPL13A* [24]. Primer sequences are shown in Table 1.

Analysis of IFN-γ production. Cell culture supernatants from CD4+ cells differentiated towards Th1 cells in the presence of SARS-CoV-2 proteins were analyzed using the Human IFN-gamma Quantikine ELISA Kit from R&D Systems (Minneapolis, MN, USA). Absorbance at 450 nm was read in a Sunrise microplate reader (Tecan, Männedorf, Switzerland).

Statistics. The results were analyzed using Friedman repeated measures ANOVA on ranks followed by Student–Newman–Keuls post hoc test. A *p*-value of 0.05 or lower was considered statistically significant. Statistical analysis was performed using SigmaStat ver.3.5 (Systat Software Inc. San Jose, CA, USA). 

## 3. Results

During the initial stage of our study, we examined whether the presence of coronavirus SARS-CoV-2 proteins S (spike) and N (nucleocapsid) influences cytokine expression in CD4+ cells activated under conditions favoring acquisition of the Th1 or Th17 phenotype. To do this, we isolated CD4+ cells from the buffy coats of healthy donors, which were subsequently cultured in the absence or presence of S and N SARS-CoV-2 proteins under Th1 and Th17 polarizing conditions for five days. As shown in Figure 1A, we observed strong induction of *IFNG* expression in cells cultured in Th1 polarizing conditions and a smaller but significant induction of *IL2* expression (Figure 1B) in CD4+ cell cultures initiated from different individual donors. Interestingly, no effect on the expression of *TNF* was observed (Figure 1C), and the expression of the master regulator of Th1 cells, *TBX21* (Figure 1D), was slightly decreased. Induction of IFN-γ was also confirmed at the protein level by ELISA (Figure 2). In striking contrast, we did not observe the same effects of S and N SARS-CoV-2 proteins on *IFNG* gene expression in CD4+ cells cultured under conditions promoting differentiation into Th17 lymphocytes (Figure 3A). The presence of viral proteins also had no effect on expression of the Th17-specific *RORγT* or *IL17A/F* genes (Figure 3B–D). These observations suggest that SARS-CoV-2 proteins selectively enhance cytokine expression in Th1 cells but not Th17 cells in healthy individuals.

To further investigate this phenomenon, we examined the effects of these proteins on in vitro differentiated Th1 cells. To do this, we cultured CD4+ cells in Th1-polarizing conditions for five days and then restimulated them with anti-CD3 and anti-CD28 in the absence or presence of SARS-CoV-2 S and N proteins for 24 h in media with no additional cytokines. Similar to CD4+ cells differentiated towards Th1 in the presence of S and N coronavirus proteins, we observed induction of *IFNG* expression (Figure 4A) in all cultures obtained from different individual donors. Interestingly, unlike initial CD4+ cells differentiated towards Th1 cells, the expression of *IL2* (Figure 4B) was not affected. This indicates that different mechanisms regulate *IL2* expression in these two cellular models, or there is an effect due to differences in the duration of exposure of the cells to these proteins (5 days vs. 24 h). 

## 4. Discussion

SARS-CoV-2 is a new virus, and we know relatively little about its mechanisms of action and interaction with the host immune system, including CD4+ lymphocytes. Similar to SARS-CoV-1 and MERS-CoV, SARS-CoV-2 disturbs the coordinated response of the immune system, leading to increased cytokine and chemokine release at local and systemic levels and, consequently, infiltration of inflammatory cells, apoptosis, and organ injury [6]. Several studies have shown the responses of T cells from COVID-19 patients to different SARS-CoV-2-derived peptides matching sequences of S and N proteins; these cellular responses included increased expression of several cytokines such as IFN-γ, IL-2, and IL-17 in CD4+ cells [25,26,27,28,29,30]. While some studies have reported that T cells from unexposed individuals did not respond to viral peptides [31], other reports observed responses to SARS-CoV-2 antigens in 20 to 60% of tested unexposed individuals [25,27]. One possible explanation for these observations is the cross-reactivity of SARS-CoV-2 antigens with common cold coronaviruses, similar to that observed previously for SARS-CoV-1 [32]. Indeed, a recent paper by Mateus et al. showed that there are memory CD4+ T cells that cross-react with SARS-CoV-2 and the common cold coronaviruses [27]. The common cold is the most common human infectious disease, and it can affect adults up to 5 times a year and children up to 10 times a year [33]. Coronaviruses HCoV-229E, HCoV-HKU1, HCoV-NL63, and HCoV-OC43 account for 8–15% of all cases, and similar to other respiratory diseases, they display seasonal patterns in detection frequency [34,35]. Until now, there has been no evidence of whether such cross-reactivity to common cold coronaviruses is beneficial, although research on influenza virus suggests that it might be [36]. 

In the present study, we show that SARS-CoV-2 proteins S and N induce *IFNG* expression in CD4+ cells differentiated towards Th1 cells and in already differentiated Th1 cells obtained from unexposed healthy individuals. This cellular response seems to be restricted to Th1 cells, as Th17 cells from the same individuals did not exhibit this response. Our observations are consistent with the hypothesis that in peripheral blood from previously unexposed individuals, there are memory CD4+ Th1 cells [37] that recognize epitopes present in S and N SARS-CoV-2 proteins. The observation that Th1 cells responded similarly to both S and N proteins is in agreement with another report [26], while a Th17-like response was not observed. This suggests that Th17-mediated immunopathology, which occurs in some patients with acute respiratory distress syndrome (ARDS) [38], is rather the effect of propagation of an invalid signal leading to activation of Th17 lymphocytes (and other cells) at later stages of the disease. The limitations of our study include the relatively low number of donors and that samples were collected in the initial phase of the pandemic (and not before the pandemic). However, when the study started, the number of COVID-19 cases in Poland was 32,821, which represented 0.085% of the country’s population; when the study ended, the number of cases was 57,876, which represented 0.15% of the country’s population. Thus, the probability that we had randomly selected even a single positive donor who did not develop antibodies [39] was rather low. Furthermore, in contrast to the cells of COVID-19 patients [25], our cells exhibited several-fold lower IFN-γ production and no *TNF* induction. Despite the mentioned limitations, our observation that all donors (Figure 1A, Figure 2, Figure 4A and DataSet S1) responded to SARS-CoV-2 antigens suggests that a significant portion of the Polish population may possess immunological memory that recognizes the SARS-CoV-2 antigens associated with Th1 lymphocytes, possibly generated during frequent common cold coronavirus infections. However, to our knowledge, there are no serological tests that would provide stronger evidence that immunological memory is due to those common cold coronaviruses. It is tempting to speculate a predisposition to developing such Th1-associated immunological memory might be linked to immunomodulatory effects of certain vaccinations, e.g., with BCG [40] or pneumococcal vaccines [41]. 

## 5. Conclusions

The ability of the immune system to develop Th1 responses against SARS-CoV-2 seems to be clinically important, since COVID-19 patients with severe disease had a significantly diminished Th1 response [42] in response to low levels of induction of IFN-γ by SARS-CoV-2 antigens [43]. This might be a potentially important observation for further population-based studies, in order to understand how the immunological status of different populations might affect the course of SARS-CoV-2 epidemics in different geographic locations. 

## Figures and Tables

**Table 1:** PCR primers used in quantitative RT-PCR.

Gene	Forward Primer	Reverse Primer
*TBX21*	5′-AGCTCACAAACAACAAGGGG-3′	5′-ATTCTGGTAGGCAGTCACGG-3′
*IFNG*	5′-CAGGTCATTCAGATGTAGCGG-3′	5′-CATGTATTGCTTTGCGTTGG-3′
*IL2*	5′-TTTTACATGCCCAAGAAGGC-3′	5′-ATGGTTGCTGTCTCATCAGC-3′
*TNF*	5′-CCAGGCAGTCAGATCATCTTCTCG-3′	5′-ATCTCTCAGCTCCACGCCATTG-3′
*RORγT*	5′-CTGCTGAGAAGGACAGGGAG-3′	5′-AGTTCTGCTGACGGGTGC-3′
*IL17A*	5′-AAACAACGATGACTCCTGGG-3′	5′-CTTGTCCTCAGAATTTGGGC-3′
*IL17F*	5′-CTTTCTGAGTGAGGCGGC-3′	5′-TGGGAACGGAATTCATGG-3′
*HPRT1*	5′-TGACACTGGCAAAACAATGCA-3′	5′-GGTCCTTTTCACCAGCAAGCT-3′
*HMBS*	5′-GGCAATGCGGCTGCAA-3′	5′- GGGTACCCACGCGAATCAC-3′
*RPL13A*	5′-CCTGGAGGAGAAGAGGAAAGAGA-3′	5′-TTGAGGACCTCTGTGTATTTGTCAA-3′

## Supplementary Materials

The following are available online at https://www.mdpi.com/2076-393X/8/4/673/s1. DataSet S1: datasets for Figure 1A, Figure 2 and Figure 4A with statistical analysis.
